# First Record of *Leishmania* (*Viannia*) sp. and High Prevalence of *Anaplasma marginale* and *Trypanosoma theileri* in Zebu Cattle from Zenú Communities in Northern Colombia

**DOI:** 10.3390/pathogens14040382

**Published:** 2025-04-15

**Authors:** Daniel Guzmán-Vásquez, Lucas Lisboa Nunes Bonifácio, Kamila Gaudêncio da Silva Sales, Rafaela Lira Nogueira de Luna, Luis Enrique Paternina Tuiran, Filipe Dantas-Torres

**Affiliations:** 1 Institute of Tropical Biological Research, Faculty of Veterinary Medicine and Zootechnics, Universidad de Córdoba, Montería 230002, Colombia; biologodanielguzman@gmail.com; 2 Instituto Aggeu Magalhães, Fundação Oswaldo Cruz (Fiocruz), Recife 50740-465, PE, Brazil; lucas_lisboa25@hotmail.com (L.L.N.B.); kamilasalesg@gmail.com (K.G.d.S.S.); rafaelalira.luna@hotmail.com (R.L.N.d.L.); 3 Department of Biology and Chemistry, Faculty of Education and Science, Universidad de Sucre, Sincelejo 700001, Colombia

**Keywords:** *Leishmania*, *Anaplasma*, *Trypanosoma*, indigenous communities, cattle

## Abstract

Colombia has the fourth largest livestock herd on the American continent. Cattle farms are expanding in Colombia, sometimes impacting traditional communities and reserves. This is especially true for the Zenú ethnic group, whose ancestral territory includes the valleys of the Sinú and San Jorge rivers, as well as the Caribbean coast around the Gulf of Morrosquillo, in the departments of Córdoba and Sucre. The present study examined the prevalence of trypanosomatids and *Anaplasma* spp. in zebu cattle grazing in seven Zenú communities in the Sucre department. Of the 110 cattle sampled, 56 (50.9%) tested positive for trypanosomatids. Forty *18S* rRNA gene sequences generated showed >99% identity with *Trypanosoma theileri,* while one sequence demonstrated 99.6% identity with *Leishmania* (*Viannia*) *braziliensis* and *Leishmania* (*Viannia*) *panamensis*; sequencing of the remaining 15 positive samples was unsuccessful. Regarding *Anaplasma* spp., 96 (87.3%) samples were positive, and the 14 *msp4* gene sequences generated displayed >99% identity with *Anaplasma marginale*. Thus, *T. theileri* and *A. marginale* were prevalent in cattle from all Zenú communities, while *Leishmania* (*Viannia*) sp. was found in a cow from the community of La Gallera. Our findings indicate that these agents are common in zebu cattle from Zenú communities, underscoring the need for preventive measures to reduce the infection burden and potential implications for cattle production in these areas.

## 1. Introduction

With over 29 million cattle, Colombia has the fourth-largest livestock herd in the Americas and ranks 14th globally [1]. The cattle industry plays a significant role in the national economy. In addition to its economic contributions, livestock farming is a vital pillar of the country’s food security, accounting for 21.8% of the agricultural gross domestic product [2]. Zebu cattle make up 95% of the bovine population in Colombia [3] and have been employed in crossbreeding systems with Colombian creole cattle breeds [4].

Zebu cattle are prevalent in various regions of Colombia. Depending on their environmental context, these cattle may encounter several parasites, including ticks, that can transmit various pathogens, such as bacteria and protozoa. For example, bacteria from the genus *Anaplasma* Theiler, 1910 and protozoa from the genus *Trypanosoma* Gruby, 1843 can cause bovine anaplasmosis and trypanosomiasis, respectively [5,6,7,8,9]. These diseases can negatively impact dairy and meat production worldwide [10,11].

The agents of bovine anaplasmosis and trypanosomiasis are prevalent in tropical and subtropical regions worldwide, being endemic to the Americas with varying levels of prevalence based on geographical areas [12,13]. For example, bovine anaplasmosis, primarily caused by *Anaplasma marginale* Theiler, 1910, poses a significant threat to livestock productivity in Latin America, with an average prevalence of 48.9% in the region [13]. In Colombia, a recent study indicated a high prevalence of 59.9% in the northeastern region [8]. A study conducted on the northern coast of Colombia found that calves typically become infected with *A. marginale* at an average age of 11 weeks [14]. This underscores that *A. marginale* is widely distributed across various regions of Colombia, posing a substantial challenge for the cattle industry.

Bovine trypanosomiasis is a widespread disease in Latin America, where cattle can become infected with *Trypanosoma vivax* Ziemann, 1905, *Trypanosoma evansi* (Steel, 1885)*,* and *Trypanosoma theileri* Laveran, 1902. These parasites are usually transmitted mechanically by tabanid flies (family Tabanidae) or *Stomoxys calcitrans* (Linnaeus, 1758) (family Muscidae) [11,15,16,17,18,19]. In Colombia, the prevalence of these parasites varies by region and cattle breed. A study conducted on farms in central and western Colombia examined the prevalence of different *Trypanosoma* species in the Casanareño, Chino Santandereano, and Sanmartinero creole breeds [7]. The overall prevalence ranged from 33.3% in Sanmartinero to 75.2% in Chino Santandereano, with *T. theileri* being the most common species. Another trypanosomatid parasite, which is rarely reported in cattle in Latin America, is *Leishmania infantum* Nicolle, 1908 [20,21], the causative agent of zoonotic visceral leishmaniasis. Nevertheless, little is known about cattle’s exposure to other *Leishmania* spp., such as *Leishmania* (*Viannia*) *braziliensis* Vianna, 1911 and *Leishmania* (*Viannia*) *panamensis* Lainson and Shaw, 1972, which are prevalent in this region.

Cattle grazing is expanding in several regions of Colombia, occasionally affecting traditional communities and reserves. This includes the Zenú people (or Senú), whose ancestral territory spans the valleys of the Sinú and San Jorge rivers, as well as the Caribbean coast around the Gulf of Morrosquillo, situated in the departments of Córdoba and Sucre. The Zenú reserve is undergoing significant changes due to several factors, including the growth of nearby cattle pastures and monoculture rice plantations. There is no information about the health status of cattle grazed in Zenú communities. This study investigated the molecular prevalence of trypanosomatids and *Anaplasma* spp. in 110 zebu cattle from various Zenú communities in Sucre.

## 2. Materials and Methods

### 2.1. Study Area and Population

**Figure 1:**
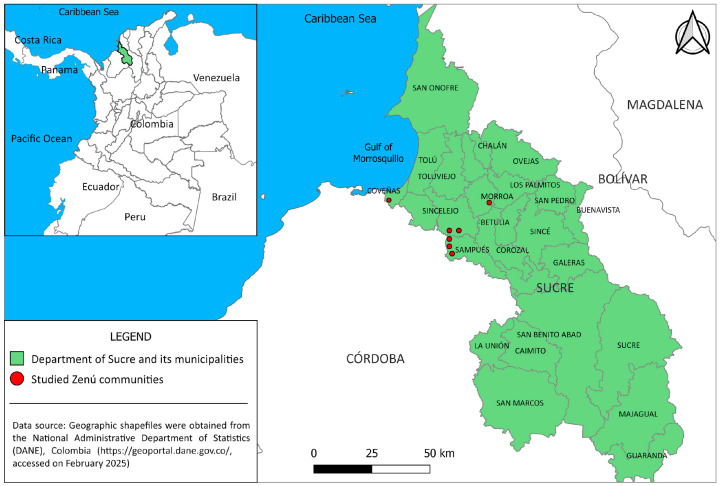
Map of Sucre, northern Colombia, showing the Zenú communities’ location. This map was created using QGIS version 3.34.15 (https://qgis.org, accessed on 12 February 2025).

From March to September 2023, a total of 110 zebu cattle (23 males and 87 females; average age = 2.9 years) from seven Zenú communities were included in this study. These animals came from the municipalities of Sincelejo, Sampués, Coveñas, and Morroa in the Sucre department (Figure 1). The age, sex, and location of each animal were recorded.

### 2.2. Blood Sampling and DNA Extraction

After obtaining consent from the holder, each animal was physically immobilized, and a 1.5 mL sample was drawn from its jugular vein. Blood samples were placed in EDTA tubes and stored at −20 °C. Subsequently, genomic DNA was extracted using the mini-prep DNA extraction method described by Afanador et al. [22]. The extracted DNA was resuspended in 50 μL of ultrapure water and kept at −20 °C until needed.

### 2.3. Detection of Trypanosomatid DNA

Trypanosomatid DNA was detected using nested polymerase chain reaction (PCR), targeting a fragment of a variable region of the *18S* ribosomal RNA gene (*18S* rRNA) across two amplification rounds. In the first round, each 25 µL reaction comprised 5 µL of a genomic DNA sample, 2.5 µL of DNA-free water, 12.5 µL of GoTaq^®^ Colorless Master Mix (Promega, Madison, WI, USA), and 2.5 µL of each primer at a concentration of 20 pmol/µL, including the following external primers: TRY927F (5′-GAAACAAGAAACACGGGAG-3′) and TRY927R (5′-CTACTGGGCAGCTTGGA-3′) [23]. The thermocycler settings were as follows: 30 cycles at 94 °C for 30 s, 55 °C for 60 s, and 72 °C for 90 s [23]. The products from the first amplification were diluted (1:10) in sterile deionized water. For the second round of PCR, 2 µL of this dilution served as the template. Each reaction had a final volume of 25 µL, which included 5.5 µL of DNA-free water, 12.5 µL of GoTaq^®^ Colorless Master Mix (Promega, Madison, WI, USA), and 2.5 µL of each primer at a concentration of 20 pmol/µL of the internal primers: SSU561F (5′-TGGGATAACAAAGGAGCA-3′) and SSU561R (5′-CTGAGACTGTAACCTCAAAGC-3′) [19]. These primers amplify a fragment of approximately 556 bp [23]. The thermal cycling conditions were set as follows: denaturation at 94 °C for 3 min, followed by 30 cycles of 94 °C for 1 min, 48 °C for 120 s, 72 °C for 2 min, and a final elongation at 72 °C for 10 min. A master mix without DNA (no template control) and a DNA sample extracted from cultured promastigotes of *L. infantum* (positive control) were included in each assay. All thermal cycling was performed using a Veriti^®^ 96-well thermal cycler (Applied Biosystems, Foster City, CA, USA).

To further investigate the identity of a *Leishmania* sp. detected in this study, we employed a PCR protocol targeting a fragment of the heat-shock protein 70 (*hsp70*) gene of *Leishmania* spp., as described elsewhere [24].

### 2.4. Detection of Anaplasma marginale

*Anaplasma marginale* DNA was detected using a PCR assay that targets the major surface protein 4 (*msp4*) gene, employing the primers msp45 F (5′-GGGAGCTCCTATGAATTACAGAGAATTGTTTAC-3′) and msp43 R (5′-CCGGATCCTTAGCTGAACAGGAATCTTGC-3′) [20]. PCR reactions were performed in a 25 μL volume, which included 12.5 μL of Gotaq^®^ colorless master mix (Promega, Madison, WI, USA), 5.5 μL of DNA-free water, 2.5 μL of each primer at a concentration of 20 pmol/μL, and 2 μL of DNA. These primers amplify a fragment of approximately 854 bp [25]. The thermal cycling conditions comprised an initial denaturation at 95 °C for 120 s, followed by 35 cycles of denaturation at 95 °C for 60 s, alignment at 60 °C for 60 s, and extension at 72 °C for 60 s, with a final extension at 72 °C for 5 min.

### 2.5. Electrophoresis, Amplicon Purification, and Sequencing

The amplification products, stained with ethidium bromide, were loaded onto a 1.5% agarose gel and visualized under UV light (L-PIX EX, Loccus, Cotia, Brazil). Amplicons of the expected size for each PCR were purified using the PureLink PCR Micro Kit (Invitrogen, Carlsbad, CA, USA), following the manufacturer’s instructions. The purified products underwent Sanger sequencing on a 3500 XL genetic analyzer (Applied Biosystems) in both directions, using the same primers as those for PCR. The sequences were trimmed (Phred quality score ≥ 20) and assembled with Geneious Prime version 2025.0.3 (Dotmatics, Boston, MA, USA). The consensus sequences were compared with those available in the GenBank database using the Nucleotide Basic Local Alignment Search Tool (BLASTn) (https://blast.ncbi.nlm.nih.gov, accessed on 8 April 2025). Representative sequences of each pathogen species identified were deposited in GenBank (accession numbers PP778348, PV361573, PV361762, PV361765, PV361766, PP777987, PV424041, and PP793891).

### 2.6. Phylogenetic Analysis and Haplotype Diversity

The consensus sequences generated in this study were aligned with sequences retrieved from GenBank using MAFFT multiple sequence alignment software version 7 [26] and trimmed in Molecular Evolutionary Genetics Analysis version 12 (MEGA12) [27] to standardize the sequence lengths. The maximum likelihood (ML) method was employed to infer the evolutionary history using IQ-TREE, with 1000 ultrafast bootstrap (UFBoot) replicates [28,29]. According to the Bayesian Information Criterion (BIC), the best-fit models identified were TIM2e+I+G4 and TPM2+F+G4 for the *18S* rRNA and *msp4* phylogenetic trees, respectively. The *18S* rRNA and *msp4* trees were rooted using the nucleotide sequences of *Leishmania amazonensis* Lainson and Shaw, 1972 (GenBank accession number: JX030087) and *Ehrlichia ruminantium* (Cowdry 1925) (GenBank accession number: BDDN01000175), respectively. The final phylogenetic tree was edited using iTOL v.7 (https://itol.embl.de/, accessed on 1 March 2025). The number of haplotypes (H), haplotype diversity (Hd), nucleotide diversity (π), and the average number of nucleotide differences (*K*) were calculated using DnaSP version 6.12.03 [30].

### 2.7. Data Analysis

The proportion of positives and the exact 95% confidence intervals (95% CI) were calculated using GraphPad QuickCalcs (http://www.graphpad.com/quickcalcs, accessed on 28 February 2025).

## 3. Results

Out of the 110 cattle examined, 56 (50.9%; 95% CI: 41.2–60.6%) tested positive for trypanosomatids, and 41 consensus sequences were successfully generated. BLASTn searches revealed that 40 sequences (sizes ranging from 432 to 542 bp) exhibited identities ranging from 99.4% to 100% (100% query coverage) with *T. theileri* sequences from various countries (GenBank accession numbers: ON063536, LC385952, KJ195881, KJ397592, ON063535, ON063534, JX178182, AJ009163, KF924255, KF924256). The remaining sequence (542 bp), obtained from a cow in the La Gallera community (municipality of Sincelejo), showed 99.5–99.6% identity with *L.* (*V.*) *braziliensis* (accession numbers: OY748421, OY748513, GQ332355, JX030135), 99.6% with *L.* (*V.*) *panamensis* (accession number: JN003595), following by 99.1% with both *L.* (*V.*) *guyanensis* (accession number: X53913) and *L.* (*L*.) *amazonensis* (accession numbers: JX030083, PP718781, PP718783). Our attempt to amplify a fragment of the *hsp70* gene of *Leishmania* spp. for species identification was unsuccessful. In total, 36.4% (95% CI: 27.4–46.1%) of the animals were positive for *T. theileri,* and 0.9% (95% CI: 0.0002–5.0%) for *Leishmania* (*Viannia*) sp. Detailed information about single and double infections is provided in Table 1.

We identified five haplotypes (Hd = 0.603, π = 0.00726, *K* = 3.035) among our 40 *T. theileri* sequences: H1 (24 sequences), H2 (two sequences), H3 (seven sequences), H4 (five sequences), and H5 (one sequence). Table 2 shows the percentage identities between these haplotypes and closely related species.

**Figure 2:**
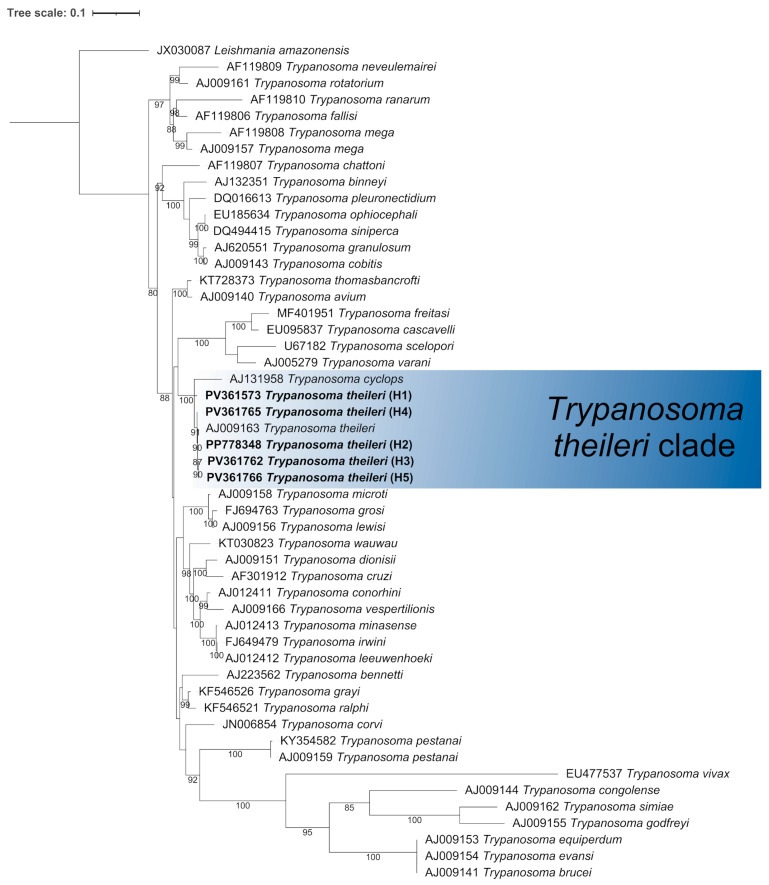
Phylogenetic reconstruction of the genus *Trypanosoma* based on partial *18S* rRNA gene sequences obtained in this study (in bold) and from GenBank. The dataset included 51 sequences and 2755 nucleotide sites. The tree was inferred using maximum likelihood with the TIM2e+I+G4 model. Bootstrap values <80% were omitted. *Leishmania amazonensis* served as the outgroup. The *Trypanosoma theileri* clade is highlighted in blue. Haplotypes (H) are indicated in parentheses.

In the phylogenetic analysis, our *T. theileri* sequences were accurately placed within the *T. theileri* clade, with strong branch support (Figure 2).

**Figure 3:**
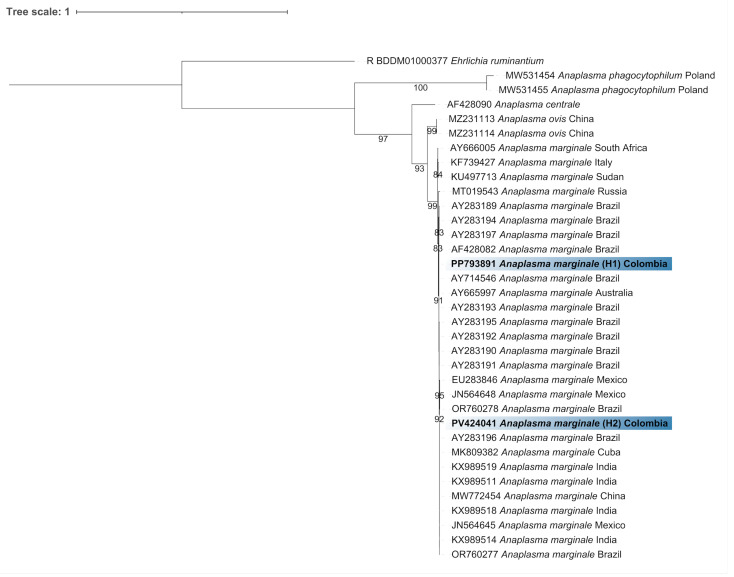
Phylogenetic reconstruction of the genus *Anaplasma* based on partial *msp4* gene sequences obtained in this study (in bold) and from GenBank. The dataset comprised 35 sequences and 3277 nucleotide sites. The tree was inferred using maximum likelihood with the TPM2+F+G4 evolutionary model. Bootstrap values < 80% were omitted. *Ehrlichia ruminantium* served as the outgroup. Haplotypes (H) are indicated in parentheses.

Regarding *Anaplasma* spp., 87.3% (96/110) of the tested bovine samples were positive, from which 14 sequences were generated (sizes ranging from 238 to 801 bp), including representatives from the various cattle communities studied. These sequences exhibited identities from 99.1% to 100% (with 100% query coverage) when compared to *A. marginale sequences* from different countries (accession numbers: MK809382, KX989519, KX989516, MK809387, CP023731, MH172467, MK140740, and MK809379). Considering our five longest *msp4* gene sequences (801 bp), we identified two haplotypes, H1 and H2 (Hd = 0.600, π = 0.00150, and *K* = 1.200), with two sequences belonging to H1 and one to H2. Based on phylogenetic analysis, our sequences were grouped with *A. marginale* sequences from various countries, separated from other *Anaplasma* spp. (Figure 3).

*Anaplasma marginale* and *T. theileri* were prevalent in cattle across all studied communities, with 31.8% of the animals being coinfected.

## 4. Discussion

We assessed the presence of trypanosomatids and *Anaplasma* spp. in bovines from Zenú communities on the Caribbean coast of Colombia. Surprisingly, one of the cattle tested positive for a *Leishmania* species from the subgenus *Viannia*, which includes several causative agents of cutaneous leishmaniasis in the Americas [31]. Detecting *Leishmania* spp. in cattle is relatively rare, although it has been reported in other countries, including *L. infantum* in Brazil [20,21,32,33]. To our knowledge, this is the first worldwide report of a *Leishmania* (*Viannia*) sp. in a cow. The sequence generated in this study showed 99.6% identity with *L.* (*V.*) *braziliensis* and *L.* (*V.*) *panamensis*, both known causative agents of cutaneous leishmaniasis in humans in Colombia [34]. The *18S* rRNA sequence obtained in this study is highly conserved and cannot distinguish between these species. Our attempts to sequence more definitive genetic markers, such as the *hsp70* gene, were unsuccessful. A study conducted in the department of Sucre, where our current research was carried out, characterized the isolates from eight patients with cutaneous leishmaniasis [34]. Using the cytochrome b (*cytb*) gene as a marker, six isolates were identified as *L*. (*V*.) *braziliensis*, one as *L*. (*V*.) *guyanensis,* and another as *L*. (*V*.) *panamensis*. Therefore, it is reasonable to speculate that the *Leishmania* (*Viannia*) sp. infecting one of the cows from the Zenú communities investigated here could belong to one of these three species, likely *L*. (*V*.) *braziliensis*, which is the most prevalent in the region.

Additionally, *L*. (*V*.) *braziliensis* has been reported in horses in Brazil [35]. Both cattle and horses are common blood sources for phlebotomine sand flies in leishmaniasis-endemic areas [36,37,38,39], which may explain their occasional exposure to *Leishmania* spp. Nonetheless, there is no evidence indicating that horses or cattle are involved in the zoonotic transmission cycle of *Leishmania* (*Viannia*) spp., such as *L*. (*V*.) *braziliensis*, whose primary reservoirs are small mammals, including wild rodents [40,41]. In this context, Bern et al. [42] found that higher numbers of cattle per 1000 m^2^ were associated with a lower risk of visceral leishmaniasis caused by *L*. (*L*.) *donovani* in Bangladesh. This suggests that cattle may have a protective effect on human exposure to phlebotomine sand fly vectors [43]. However, this hypothesis may not apply to all epidemiological scenarios and requires further research.

As anticipated from previous studies conducted across various regions of Colombia [5,6,7], *T. theileri* was prevalent in the Zenú communities studied. The positivity observed here was comparable to that reported in other areas of Colombia, with prevalence varying from 24.0% to 59.6% [5,6,7]. This variation may be related to local factors, though comparing these studies is challenging due to methodological differences. Although *T. theileri* is generally considered to have low pathogenicity in cattle [19], some animals may exhibit signs such as fever, anemia, swollen lymph nodes, and decreased hemoglobin concentration [44,45,46,47]. Additionally, a study in Colombia found that *T. theileri* was more frequently detected in cattle with anemia than those with normal packed cell volume [5].

Furthermore, we identified five haplotypes in this study, suggesting moderate genetic diversity among *T. theileri 18S* rRNA gene sequences from Colombia. A previous study in Colombia identified the genotypes IA and IIB [6], which are found globally [6]. A more recent study in Ecuador reported genotypes closely related to IC, IB, and IIB, which are present in countries such as Brazil, Venezuela, and Colombia [19]. Additional genetic markers (e.g., cathepsin L-like gene and internal transcribed spacer) would be needed to explore the genotypes and lineages of *T. theileri* circulating in cattle from the Zenú communities studied.

*Anaplasma marginale* was found in over 80% of the cattle in the Zenú communities studied. This bacterium can be transmitted to cattle through tick bites and mechanically by biting flies or blood-contaminated surfaces [48]. For example, reusing syringes—a common practice in many cattle herds—poses a risk for *A. marginale* transmission among cattle [49,50]. Syringes are frequently shared while administering vitamins to the cattle in the Zenú communities. This practice can perpetuate the high infection rates of *A. marginale* and hinder the livestock development of these communities. Among our longest *msp4* gene sequences, we identified two haplotypes that have been identified worldwide, including in other Latin American countries [49,50].

The high positivity rates for both *T. theileri* and *A. marginale* indicate that co-infections may be common in cattle from the studied Zenú communities. In fact, 31.8% of the animals were co-infected, which is similar to findings in the Colombian departments of Antioquia and Arauca, where 20.8% and 34.0% of the cattle were co-infected, respectively [5]. Further studies are needed to evaluate the clinical significance of co-infections in cattle living in Zenú communities.

## 5. Conclusions

We report the first detection of *Leishmania* (*Viannia*) sp. in a cow, highlighting the need for further research to investigate the clinical significance of this infection in cattle and to characterize the species involved accurately. Additionally, we detected a high frequency of infection by *A. marginale* and *T. theileri*, with over 30% of the animals being co-infected. Our findings demonstrate that *A. marginale* and *T. theileri* are prevalent in cattle from Zenú communities, emphasizing the necessity of implementing preventive measures to reduce the infection burden and its potential implications for cattle production in these communities.

## Figures and Tables

**Table 1:** Positivity for different pathogens in zebu cattle from Zenú communities.

Municipality	Community	*n*	*T. theileri* ^1^	*Leishmania* (*Viannia*) sp.	*Anaplasma marginale* ^2^	*T. theileri* + *A. marginale*	*A. marginale* + *Leishmania* (*V.*) sp.	Non-Infected
Sincelejo	La Gallera	9	0	0	4	2	1	2
	San Martin	19	1	0	9	6	0	3
Sampués	Bossa Navarro	20	1	0	11	7	0	1
	Siloe	10	1	0	4	5	0	0
	Escobar Arriba	3	0	0	1	1	0	1
Coveñas	Bellavista	27	1	0	15	10	0	1
Morroa	Morroy	22	1	0	16	4	0	1
Total (%)		110	5 (4.5%)	0 (0%)	60 (54.5%)	35 (31.8%)	1 (0.9%)	9 (8.2%)

^1^ Considering that different *Trypanosoma* species have been reported in cattle in Colombia, we have included only the 40 *Trypanosoma* PCR-positive samples confirmed by sequencing. ^2^ Only 14 *A. marginale msp4* gene sequences were generated, but we assume all *Anaplasma* PCR-positive samples were positive for the species *A. marginale*.

**Table 2:** Percentage identity among *18S* rRNA haplotypes identified in this study and closely related species.

Haplotypes (GenBank Accession Numbers)	Percentage Identity with *T. theileri* and Related Species, as Determined by BLASTn
H1 (PV361573)	100% with of *T. theileri* (KF924257, JX853185, JX178185), 99.4% with *T. minasense* (PQ490351), and 98.3% with *T. melophagium* (OR921420)
H2 (PP778348)	100% with of *T. theileri* (JX178181, PP778348, KF924254), 99.4% with *T. melophagium* (OR921420, PP124575), 99.3% with *T. trinaperronei* (MN752212)
H3 (PV361762)	99.8% with *T. theileri* (ON063535), 99.1% with *T. melophagium* (OR921420, PP124575), and 99.1% with *T. trinaperronei* (MN752212)
H4 (PV361765)	100% with *Trypanosoma* sp. (clade ThI, OL856000, OL855998, MK156791), 99.8% with *T. theileri* (JX178181), 99.6% with *T. melophagium* (OR921420, PP124575), and 99.4% with *T. trinaperronei* (MN752212)
H5 (PV361766)	99.8–100% with *T. theileri* (ON063535, JX178181, KF924254, JX178182), 99.3% with both *T. melophagium* (OR921420, PP124575) and *T. trinaperronei* (MN752212)

## Data Availability

All data supporting the conclusions of this study are in the manuscript. The *18S* rRNA and *msp4* gene representative sequences generated herein have been deposited in GenBank (accession numbers: PP778348, PV361573, PV361762, PV361765, PV361766, PP777987, PV424041, and PP793891).

## Abbreviations

The following abbreviations are used in this manuscript:

*A.*	*Anaplasma* (genus)
BLASTn	Nucleotide Basic Local Alignment Search Tool
*L.*	*Leishmania* (genus)
LD	Linear dichroism
*msp4*	Major surface protein 4
rRNA	Ribosomal ribonucleic acid
*T.*	*Typanosoma* (genus)
*V.*	*Viannia* (subgenus)
